# Reconstruction for 3D PET Based on Total Variation Constrained Direct Fourier Method

**DOI:** 10.1371/journal.pone.0138483

**Published:** 2015-09-23

**Authors:** Haiqing Yu, Zhi Chen, Heye Zhang, Kelvin Kian Loong Wong, Yunmei Chen, Huafeng Liu

**Affiliations:** 1 Department of Optical Engineering, Zhejiang University, Hangzhou, Zhejiang, China; 2 Shenzhen Institutes of Advanced Technology, Shenzhen, Guangdong, China; 3 School of Computer Science and Software Engineering, The University of Western Australia, Crawley, Australia; 4 Department of Mathematics, University of Florida, Gainesville, United States of America; Beijing University of Technology, CHINA

## Abstract

This paper presents a total variation (TV) regularized reconstruction algorithm for 3D positron emission tomography (PET). The proposed method first employs the Fourier rebinning algorithm (FORE), rebinning the 3D data into a stack of ordinary 2D data sets as sinogram data. Then, the resulted 2D sinogram are ready to be reconstructed by conventional 2D reconstruction algorithms. Given the locally piece-wise constant nature of PET images, we introduce the total variation (TV) based reconstruction schemes. More specifically, we formulate the 2D PET reconstruction problem as an optimization problem, whose objective function consists of TV norm of the reconstructed image and the data fidelity term measuring the consistency between the reconstructed image and sinogram. To solve the resulting minimization problem, we apply an efficient methods called the Bregman operator splitting algorithm with variable step size (BOSVS). Experiments based on Monte Carlo simulated data and real data are conducted as validations. The experiment results show that the proposed method produces higher accuracy than conventional direct Fourier (DF) (bias in BOSVS is 70% of ones in DF, variance of BOSVS is 80% of ones in DF).

## Introduction

Positron emission tomography (PET) is a nuclear medicine imaging method based on coincidence detection of photon pairs emitted from positron annihilation events [1]. Apart from operating in two-dimensional (2D) data acquisition, PET are often used in three-dimensional (3D) mode, where the interplane septa are removed. The 3D data, in contrast with the 2D data acquired, approximate line integrals of the radioactive tracer distribution along all possible lines of response (LOR’s) which are not restricted to transaxial planes [2–4]. The 2D acquisition to 3D acquisition leads to a significant improvement of the scanner sensitivity, due to the increased number of measured line of response [5–7].

In 3D PET, the amount of data collected by scanner is extremely large, therefore, the focus in reconstruction has been largely on the reduction of the computation cost. To date, significant improvements have been achieved by using the two following approaches. There have been considerable efforts aimed at speeding iterative reconstruction methods with a combination of computer acceleration techniques (e.g. graphics processing unit) [8, 9], and intelligent coding (e.g. precomputing factors or implementing [3, 10] the calculations of the system matrix in the reconstruction process [11–14]). A viable alternative to the above method is a class of approaches known as direct Fourier (DF) strategy, which has long been studied by many researchers [15]. The primary emphasis has been on rebinning the 3D data in plane integrals or in a 2D data set [16–18], and the 3D projection algorithm (3DRP) [19], and the direct Fourier reconstruction with Fourier reprojection (3D-FRP) [20], and the Fourier-wavelet restoration technique [21].

While direct Fourier method is fast and easy to implement [22–25], the reconstruction accuracy suffers from performance limitations due to the fact that the Fourier basis cannot adequately represent spatially inhomogeneous data, like that typically found in the images [26, 27]. For example, tumors or cancerous tissue, because they absorb most of the fluorine radioisotope, the intensity values of these regions are larger than surrounding materials [28, 29]. In the case, the tumors in the images that are reconstructed by direct Fourier method tends to be blurred and illegible [30, 31]. On the other hand, for the organisms imaged by PET, including tumors, their intensity values are homogeneous within that region, i.e., locally piece-wise constant [32–35]. This induces us to incorporate the TV regularization into DF framework to improve the reconstruction accuracy. Total variation was first proposed by Rudin, Osher and Fatemi (ROF) [36]. Unlike other regularization methods [37–39], it avoids over-smoothing the inhomogeneous region by minimizing the gradient field of the image. To be precise, TV preserves the locally piece smooth region but also encourages sharp variations near the edges [40–44].

Thus, the problem of reconstruction can be formulated to be an optimization problem, whose objective function consists of TV norm of the reconstructed image and the data fidelity term measuring the consistency between the reconstructed image and sinogram. This paper applies BOSVS [45] which called the Bregman operator splitting algorithm with variable step size to solve the optimization problem [46, 47]. The algorithm uses the variable Barzilai-Borwein (BB) step instead of the fixed BB step used in original Bregman Operator Splitting (BOS) algorithm, the stepsize rule starts with a inial value, and increases the nominal step until termination condition is satisfied. By combining a variable splitting and the alternating direction method of multipliers (ADMM) with a Barzilai-Borwein approximation to the Hessian, the convergence of reconstruction turns out to be faster.

We evaluate the quality of the proposed method by using Monte Carlo simulated data and real patient data. After rebinning the 3D data, we compare performance in terms of contrast recovery, noise performance, performance on detecting a small target or regions, initialization issues and robustness.

## Methods

The whole framework includes two parts: 3D data rebinning and 2D image reconstruction. This section is organized as follows: First of all, the detail about 3D data in PET scanner is given, and then we adopt the Fourier rebinning algorithm (FORE) to sort the 3D data into a stack of ordinary 2D data sets as sinogram data. Then, we introduce the total variation (TV) constrained direct Fourier reconstruction schemes. At last, we apply BOSVS to solve the resulting problem.

### 3D PET Imaging Model

#### 3D Data for PET Scanner

**Fig 1:**
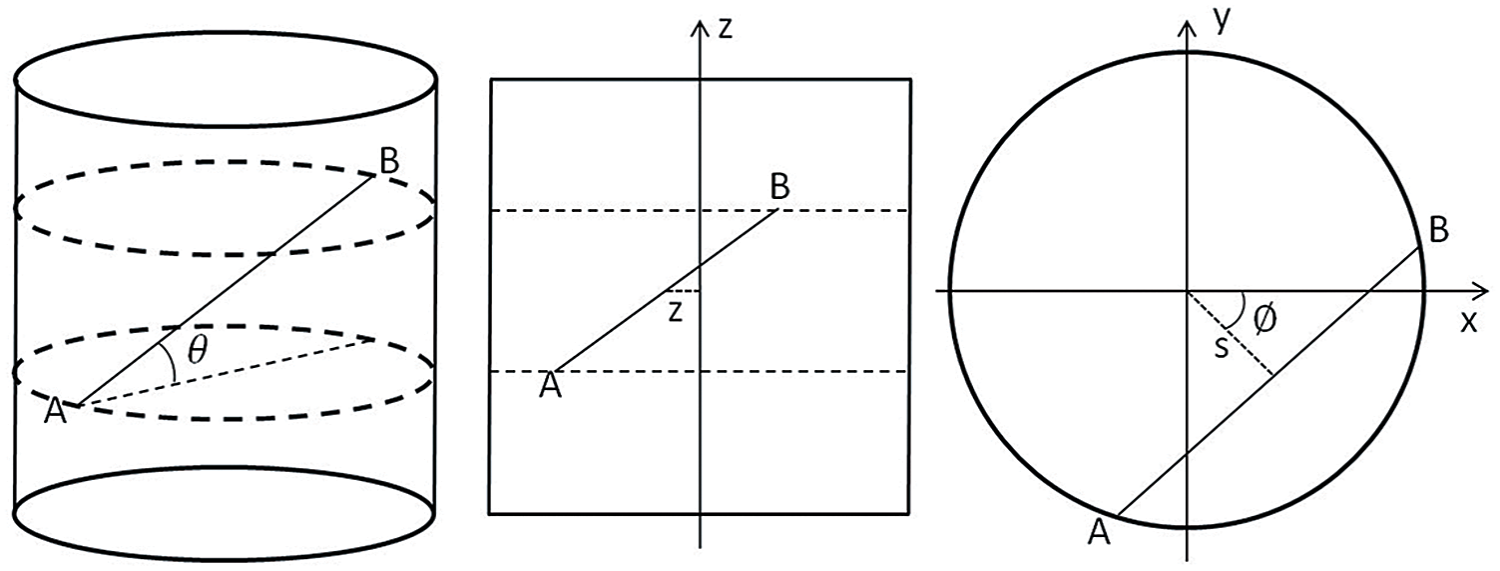
Geometry of a cylindrical PET scanner. Full view (left) about LOR’s between detector A and detector B. Longitudinal section (middle) with the axial variable z. Transaxial view (right) with the standard sinogram variables *s* and *ϕ*.

Our study uses the same geometry and data parametrization as those in [5] for the approximate Fourier rebinning algorithm FORE. As shown in Fig 1, the scanner is a truncated cylinder with radius *R*(*R* = *r*_*f*_) and length *L*, with its axis along the *z* axis. Line integrals of the tracer distribution *f*(*x*, *y*, *z*) are measured for all LOR’s connecting detector *A* and detector *B* on the surface of the cylinder. After normalization by a factor $1/\sqrt{\left(1+\delta^{2}\right)}$, these data are given by

$$\begin{array}{c} p\left(s,\phi ,z,\delta \right)=\int_{-\infty }^{+\infty }dlf\left(s\cos\phi -l\sin\phi ,s\sin\phi +l\cos\phi ,z+l\delta \right), \end{array}$$ (1)

where we have simplified notations by replacing the limits $\pm \sqrt{r_{f}^{2}-s^{2}}$ of the integral over *l* by ±∞, using the fact that *f* = 0 outside the cylinder of radius *r*_*f*_. The variable *z* is the axial coordinate of the point mid-way between detector *A* and detector *B* and the axial angular variable is *δ* = tan*θ* where *θ* is the angle between the LOR’s and the transaxial plane [5, 48].

#### Rebinning 3D Data into 2D

**Fig 2:**
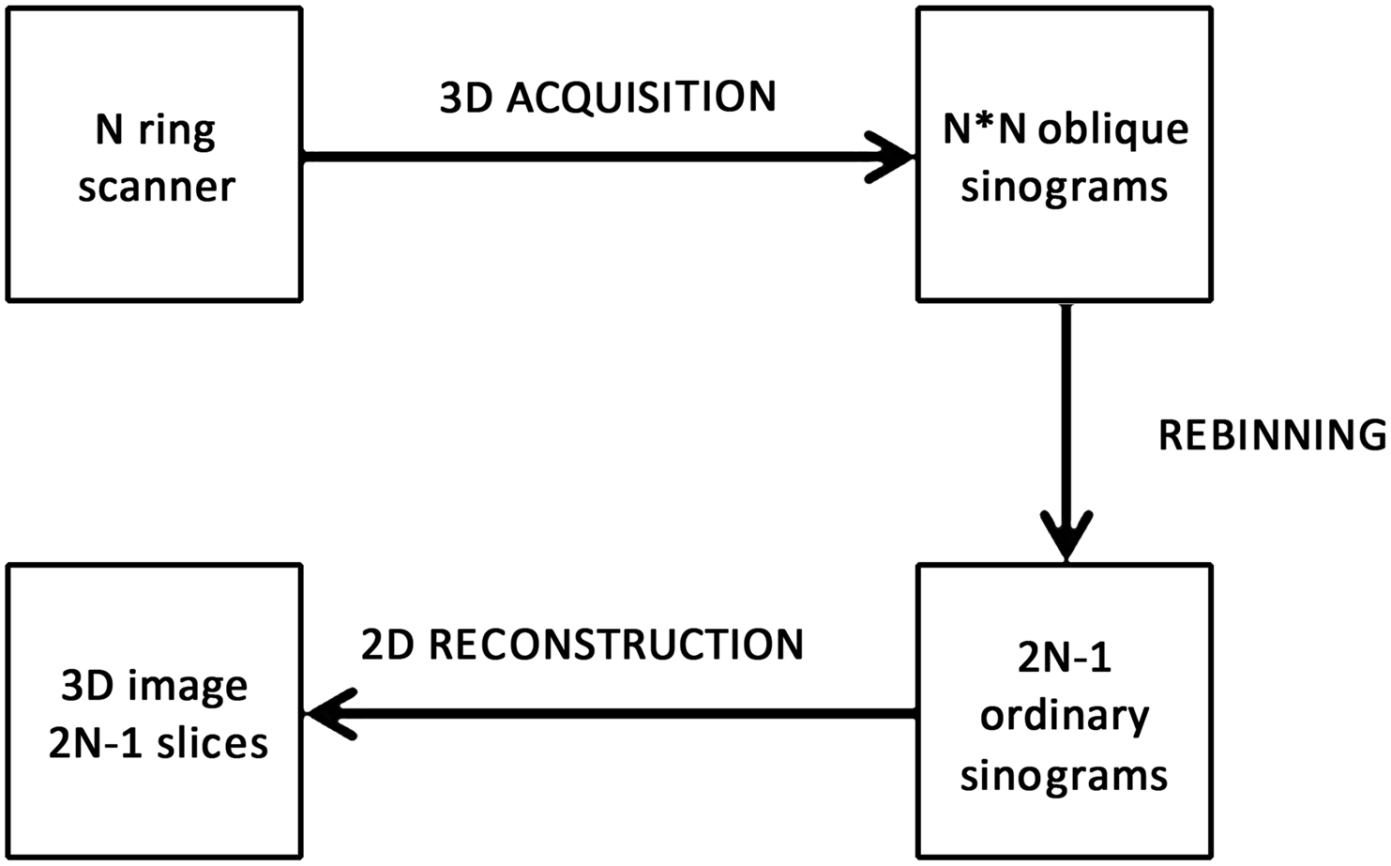
The principle of a rebinning algorithm. For multiring scanners, the oblique sinograms by 3D acquisition can be rebinned to several ordinary sinograms of N direct slices lying in the plane of the N detector and N-1 cross slices lying between adjacent detector rings.

The principle of a rebinning algorithm is summarized in Fig 2. For a fixed pair (*z*, *δ*), the set of data is referred to as an oblique sinogram. Each oblique sinogram is parameterized with the standard variables *s* and *ϕ*: *s* is the signed distance between the *z* axis and the LOR’s, *ϕ* is the angle between the *x* axis and the projection of the LOR’s onto a transaxial plane. The ranges of these variables are −*r*_*f*_ ≤ *s* ≤ *r*_*f*_ and 0 ≤ *ϕ* ≤ 2*π*.

When a scanner is operated in 2D mode, the measured LOR’s are confined to lie in a transaxial plane, so that a 2D data set can be also described by Eq (1) but with *δ* = 0

$$\begin{array}{c} p{\left(s,\phi ,z\right)}_{2D}=p\left(s,\phi ,z,0\right) \end{array}$$ (2)

These 2D data contain, for each slice *z*, one ordinary (or direct) sinogram which can be independently reconstructed by 2D reconstruction methods. Not surprisingly, this slice by slice reconstruction of a three-parameter data set is considerably faster than the reconstruction of the four-parameter 3D data set with the 3D direct reconstruction algorithm such as 3DRP.

This observation suggests an alternative approach to 3D reconstruction, in which the 3D data are not directly reconstructed, but serve to estimate 2D data from which the image can then be recovered using any 2D reconstruction algorithm such as DF. Therefore, we use the FORE(Fourier rebinning algorithm) as a method to estimate *p*_2*D*_(*s*, *ϕ*, *z*) from *p*(*s*, *ϕ*, *z*, 0) [5].

### 2D PET Imaging Model

DF methods follow directly from the ideal procedure suggested by the central slice theorem applied to the discrete case. And in a real case, only a finite number of projection are taken, giving a finite number of *F*(*u*, *v*) samples located on a uniformly distributed polar grid for the central slice theorem.

Thus, in order to obtain an approximation of *f*(*x*, *y*) by 2D inverse Fourier transform of *F*(*u*, *v*), we have first to interpolate in Fourier domain from the radial points to the points on a Cartesian grid. Basically, direct Fourier reconstruction methods are three steps methods: First, 1D discrete Fourier transform (through FFT algorithm) of the parallel projections taken at angles *θ*_1_, *θ*_2_, ⋯, *θ*_*M*_. Then, after the interpolation from Polar to Cartesian grid, the process is completed by applying 2D inverse Fourier transform (using FFT).

**Fig 3:**
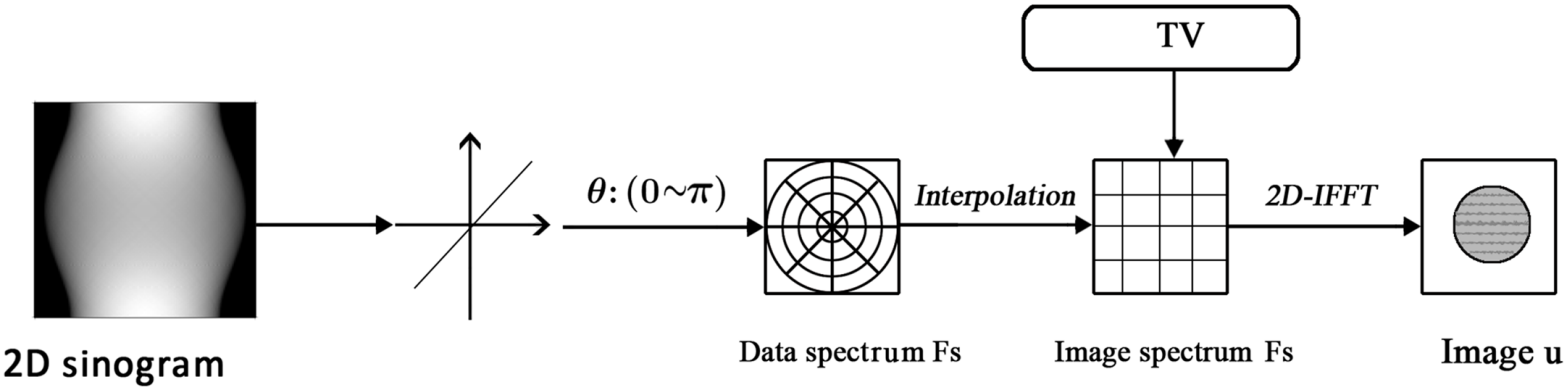
Flowchart of the proposed methods. It is illustrated for the introduction of total variation before 2D inverse FFT procedure of traditional direct Fourier method.

Unfortunately, the result of such a straight method suffer from artifacts due to interpolation in Fourier plane and to aliasing (since function *f*(*x*, *y*) is not band limited). Nevertheless, due to its lower computational complexity *O*(*N* ^2^log*N*), compared to that of the widespread Filtered Back Projections *O*(*N* ^3^), the method has been object of research and various techniques has been proposed in order to improve its performance [15]. Likewise, our proposed model will be based on the three steps and a generalized flowchart can be found in Fig 3.

### Problem Formulation

Total variation regularization is a recently image processing techniques, which has been shown to be very successful in many image processing applications such as PET and MRI [49]. And recent research has confirmed that for image restoration the use of total variation (TV) regularization instead of the *l*_1_ term makes the recovered image quality sharper by preserving the edges or boundaries more accurately, which is essential to characterize images. The advantages of TV minimization stem from the property that it can recover not only sparse signals or images, but also dense staircase signals or piecewise constant images.

As was commonly known, the fundamental equation for Fourier transform can be shown as:

$$\begin{array}{c} Fu=f, \end{array}$$ (3)

where *F* represents the Fourier transform, *f* is the spatial frequency information matrix, *u* is the reconstructed image. Thus the problem can be formulated as:

$$\begin{array}{c} \min_{u}\frac{1}{2}{∥Fu-f∥}_{2}^{2}+\alpha {∥u∥}_{TV}. \end{array}$$ (4)

Here *α* is the weighting factor of TV, and following the standard treatment we will vectorize an two-dimensional image *u* into one-dimensional column vector, *i.e.* *u* ∈ *C* ^*N*^, where *N* is the number of pixels of the image *u*. Then, the (isotropic) TV norm is defined by

$$\begin{array}{c} {∥u∥}_{TV}=\int_{\Omega }\left|Du\right|=\sum_{i=1}^{N}∥D_{i}u∥, \end{array}$$ (5)

where for each *i* = 1, ‥, *N*, *D*_*i*_ ∈ *R* ^2 × *N*^ has two nonzero entries in each row corresponding to finite difference approximations to partial derivatives of *u* at the *i*-th pixel along the coordinate axes.

### Solution

We introduce BOSVS to numerically solve the total variation based image reconstruction problem (4), which uses variable splitting method to reduce computational cost and the Barzilai-Borwein step size selection method is adopted for much faster convergence. [45]

Firstly, we introduce auxiliary variables *w*_*i*_ to transform *D*_*i*_ out of the non-differentiable norms

$$\begin{array}{ccc} & & \min_{w,u}\left\{\alpha \sum_{i}∥w_{i}∥+\frac{1}{2}{∥Fu-f∥}^{2}\right\},\\ & & \;w_{i}=D_{i}u,\forall i=1,\cdots ,N, \end{array}$$ (6)

which is clearly equivalent to the original problem (4) as they share the same solutions *u*. Then, we define *G*(*u*) as $G(u)=\frac{1}{2\alpha }\|Fu-f{\|}_{2}^{2}$, the augmented Lagrangian can be defined as:

$$\begin{array}{c} \Phi \left(w,u;\lambda \right)={∥w∥}_{2}+G\left(u\right)-<\lambda ,w-Du>+\frac{\beta }{2}{∥w-Du∥}^{2} \end{array}$$ (7)

where *λ* ∈ *C* ^2*N*^ is a Lagrange multiplier, and *β* is the penalty factor. The method of multiplier iterates the minimizations of Lagrangian *L* in Eq (7) with respect to (*w*, *u*) and the updates of the multipliers *λ*

$$\begin{array}{c} u^{k+1}=\arg\min_{u}\Phi \left(u,w^{k},\lambda^{k}\right), \end{array}$$ (8)

$$\begin{array}{c} w^{k+1}=\arg\min_{w}\Phi \left(u^{k+1},w,\lambda^{k}\right), \end{array}$$ (9)

$$\begin{array}{c} \lambda^{k+1}=\lambda^{k}-\beta \left(w^{k+1}-Du^{k+1}\right). \end{array}$$ (10)

Thus, the problem divides into several subproblems, which can be efficiently solved.

For *w* subproblem, it can be get from

$$\begin{array}{c} \min_{w}{∥w∥}_{2}+\frac{\beta }{2}{∥w-Du^{k}-\frac{\lambda^{k}}{\beta }∥}^{2}. \end{array}$$ (11)

Problem (11) has a closed form solution, 1-D Shrinkage can be applied, which for given vector *b* and scalar *μ* > 0,

$$\begin{array}{c} \min_{z}{∥z∥}_{2}+\frac{1}{2\mu }{∥z-b∥}^{2} \end{array}$$ (12)

has solution shrinkage operator

$$\begin{array}{c} S_{\mu }\left(b\right):=\max\left\{∥b∥-\mu ,0\right\}\frac{b}{∥b∥}. \end{array}$$ (13)

For *u* subproblem,

$$\begin{array}{c} u^{k+1}=\arg\min_{u}\left\{G\left(u\right)+\frac{\beta }{2}{∥Du-w^{k+1}-\frac{\lambda^{k}}{\beta }∥}^{2}\right\}. \end{array}$$ (14)

Using Bregman operator splitting to *G*(*u*) [45]:

$$\begin{array}{c} G\left(u\right)\approx G\left(u^{k}\right)+<\nabla G\left(u^{k}\right),u-u^{k}>+\frac{1}{2\delta }{∥u-u^{k}∥}^{2}, \end{array}$$ (15)

where *δ* is a constant step size. Then we drop constants in updating *u* ^*k*+1^ and complete square, as a result *u* can be written as below:

$$\begin{array}{ccc} u^{k+1} & = & \arg\min_{u}\frac{1}{2\delta }{∥u-u^{k}+\delta \nabla G\left(u^{k}\right)∥}^{2}\\ & & +\frac{\beta }{2}{∥Du-w^{k+1}-\frac{\lambda^{k}}{\beta }∥}^{2}. \end{array}$$ (16)

Take the Barzilai-Borwein step size method in the selection of step size: Using Hessian information in the selection of the step size *δ* by letting $\frac{1}{\delta_{k}^{BB}}=\nabla^{2}G(u^{k})$. Then we have

$$\begin{array}{c} \frac{u^{k}-u^{k-1}}{\delta_{BB}^{k}}=\nabla^{2}G\left(u^{k}\right)\left(u^{k}-u^{k-1}\right)\approx \nabla G\left(u^{k}\right)-\nabla G\left(u^{k-1}\right). \end{array}$$ (17)

The step size *δ* isn’t a constant any more, which can reduce computational cost and accelerate the convergence. The BB step size can be written as below:

$$\begin{array}{c} \frac{1}{\delta_{k}^{BB}}=\nabla^{2}G\left(u^{k}\right)\approx \frac{\left\langle \nabla G\left(u^{k}\right)-\nabla G\left(u^{k-1}\right),u^{k}-u^{k-1}\right\rangle }{{∥u^{k}-u^{k-1}∥}^{2}}. \end{array}$$ (18)

It converges if $\delta \leq \frac{1}{L_{G}}$ (*L*_*G*_: Lipschitz constant for ∇*G*) as proved in [47]. In our experiment, the initial value of *δ* is chosen to be 0.8 and the iteration is stopped when *δ* < 10^−5^.

Finally, *u* can be calculated as below:

$$\begin{array}{ccc} u^{k+1} & = & \arg\min_{u}\frac{1}{2\delta_{k}^{BB}}{∥u-u^{k}+\delta_{k}^{BB}\nabla G\left(u^{k}\right)∥}^{2}\\ & & +\frac{\beta }{2}{∥Du-w^{k+1}-\frac{\lambda^{k}}{\beta }∥}^{2}. \end{array}$$ (19)

The computation time for a single slice with a size of 192 by 192 pixels and iterating 100 times turns out to be 1.297345 seconds.

## Results

In order to demonstrate the performance of our proposed method, we design two groups of experiments using Monte Carlo simulated data and real data respectively. All the codes in the following experiments are implemented in Matlab R2011a (MathWorks Corporation, USA) and run in a desktop computer with i5 Intel Core CPU and 6 GB memory.

### Monte Carlo Simulation

#### PET Simulation Setup

Monte Carlo simulations can provide a relatively accurate reference for the development and assessment of new image reconstruction algorithms. Simulations in our study are performed using toolbox GATE. The simulated PET scanner is Hamamatsu SHR22000 and the phantoms chosen here for simulations are Zubal thorax phantom [50] and Hoffman brain phantom. The simulation outputs are stored in sinogram. Before reconstruction, random correction, normalization correction, attenuation correction and scatter correction are performed properly. The sinogram of brain phantom are reconstructed as images of size 64*64 and sinogram of zubal phantom are reconstructed as images of size 128*128.

#### Reconstruction Results Comparison of Different Methods

**Fig 4:**
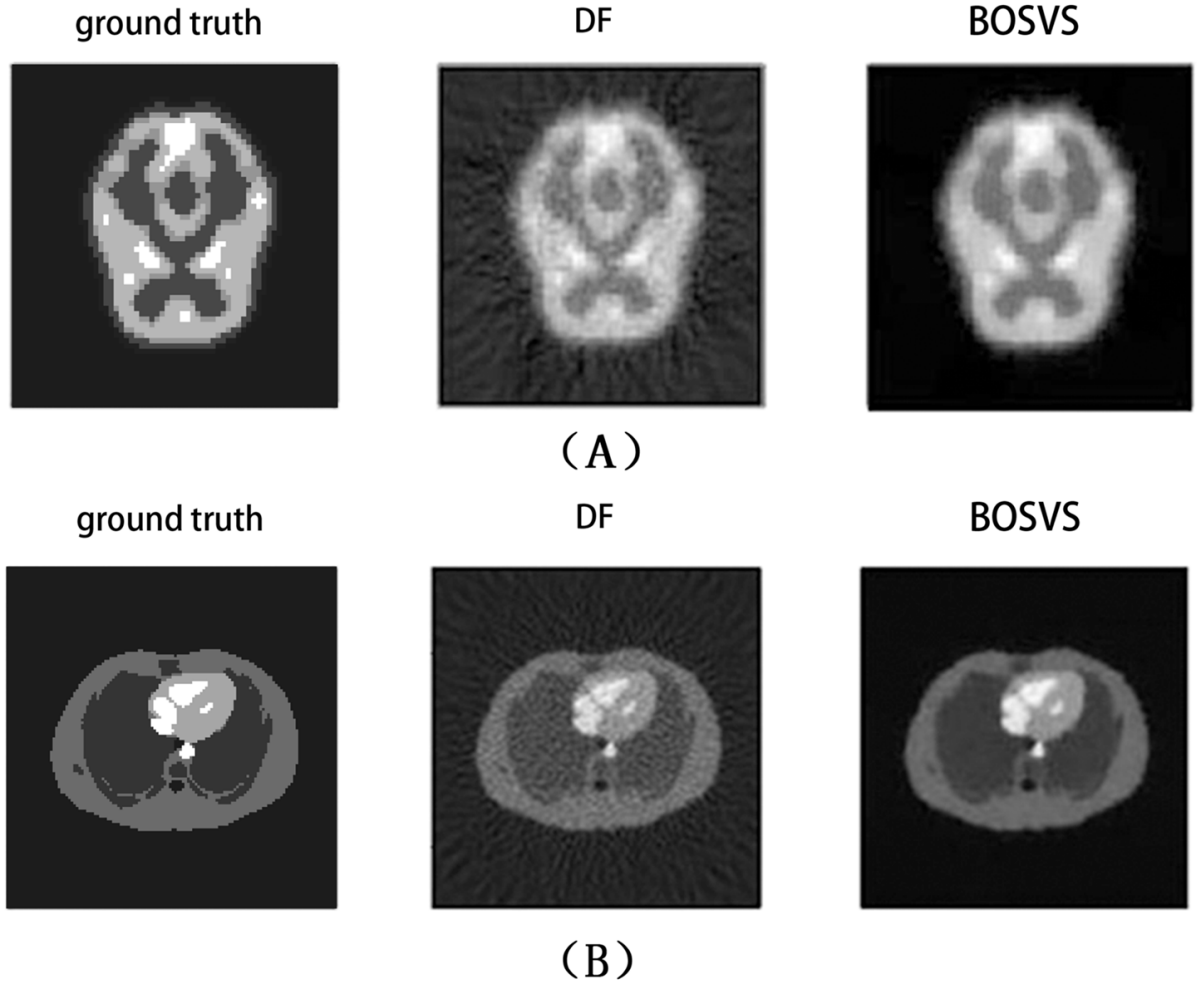
Reconstructed results of the center slice. A central transaxial slice from (A) brain phantom data, (B) zubal phantom data. Left: the ground truth. Middle: reconstructed with DF. Right: reconstructed with BOSVS.

**Fig 5:**
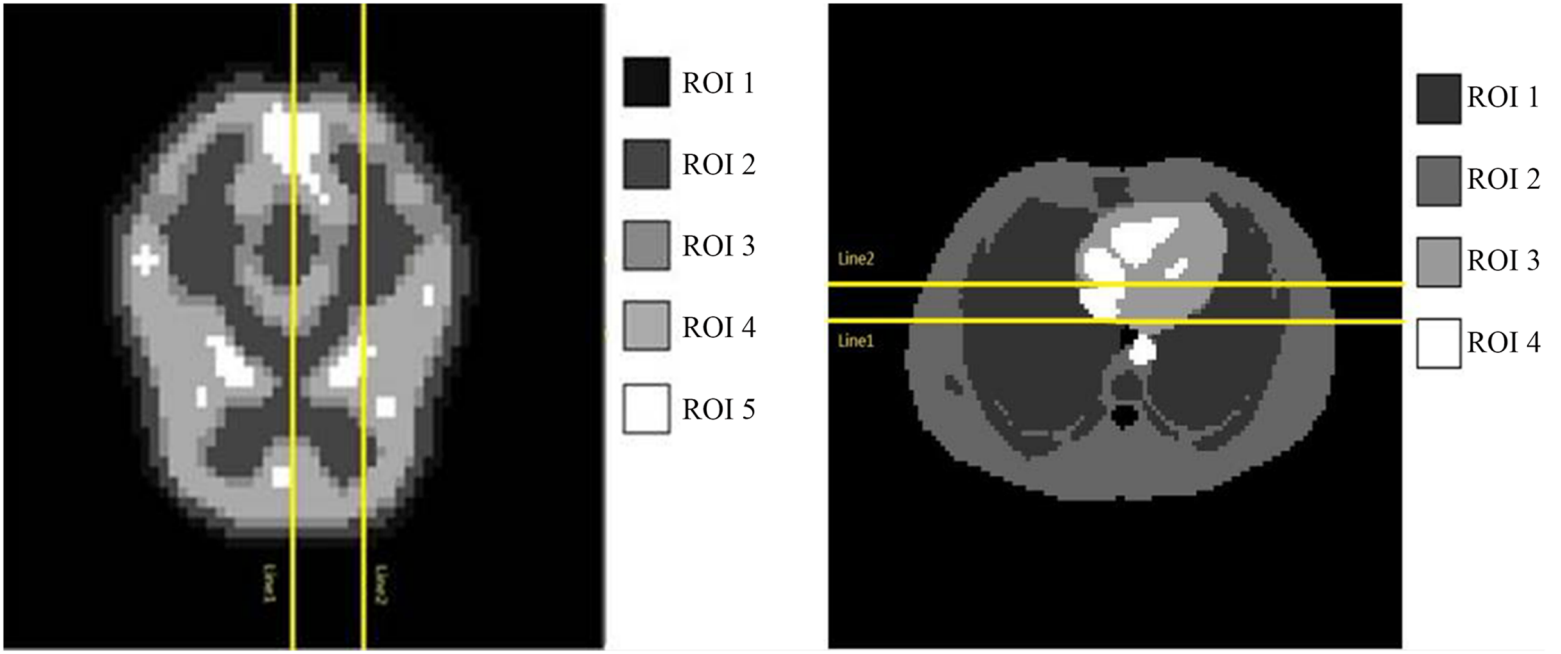
The regions of phantom and profile lines. The yellow lines mark the lateral displacement profiles. The colorful blocks demonstrate the regions of interest in the phantom.

**Fig 6:**
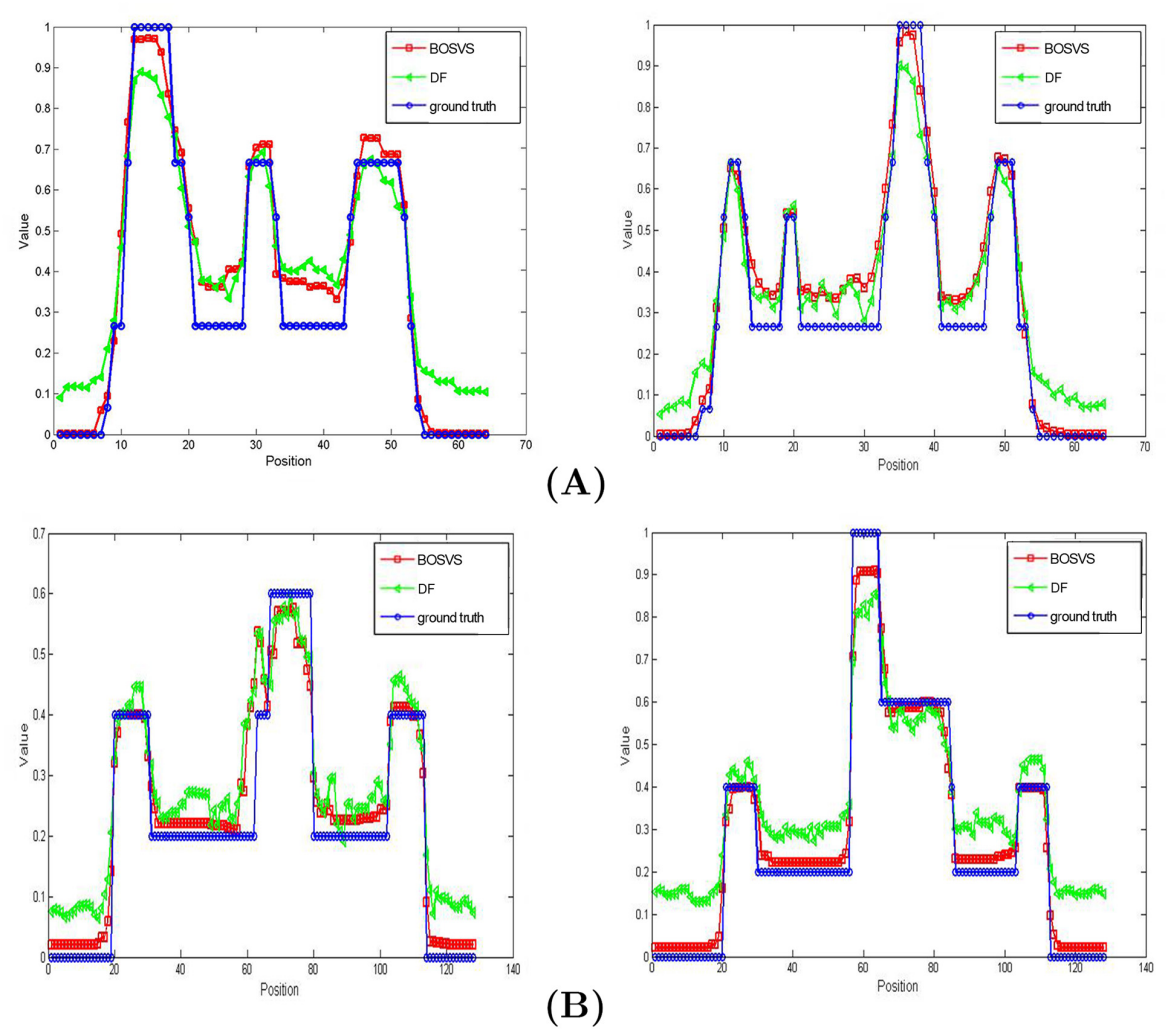
Profiles of reconstruction results. Reconstruction profiles through the marked lines in Fig 5 containing large hot features. Note that all data have been normalized.

**Fig 7:**
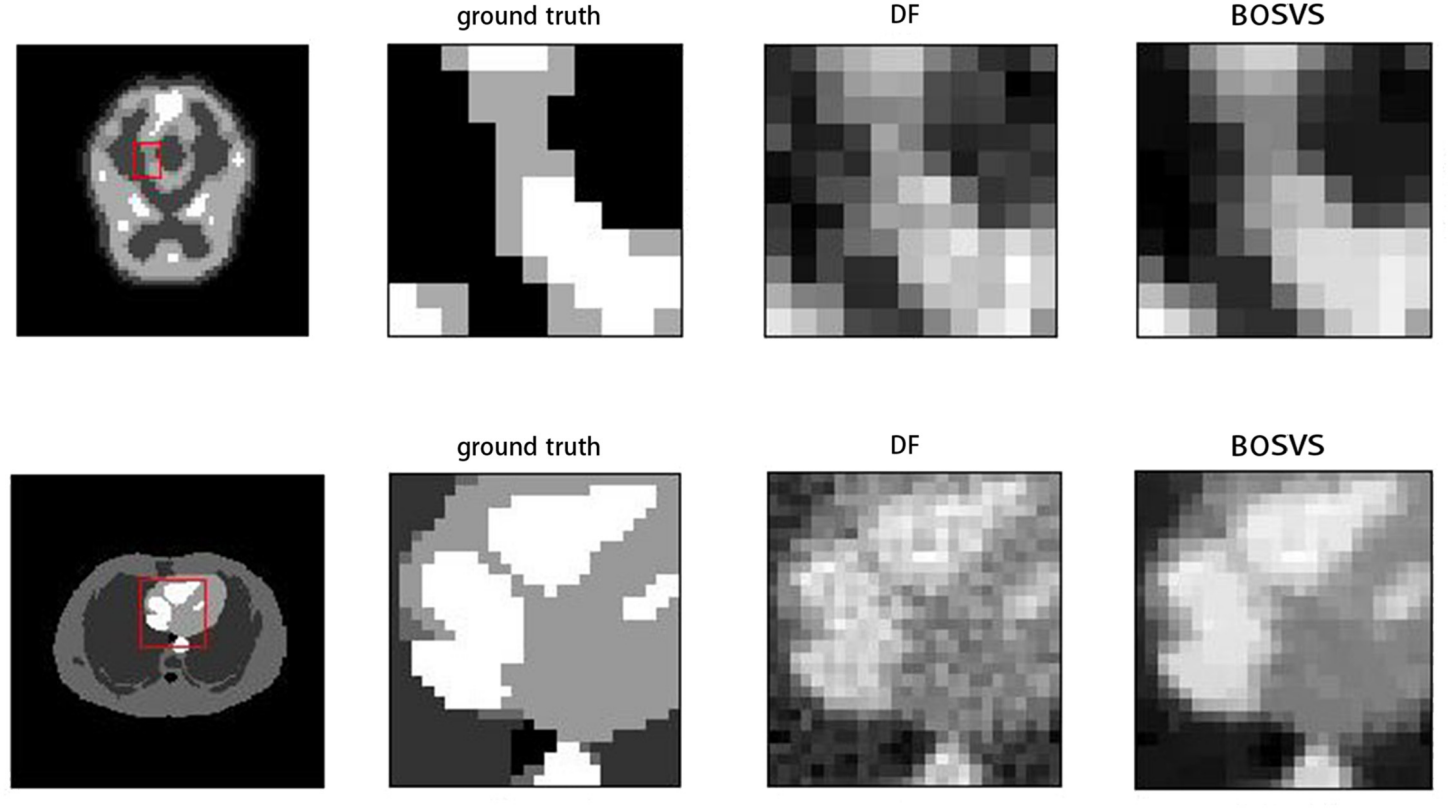
Selected area of reconstruction results. The first row gives the results using brain phantom data, left: reference image with red rectangle indicating the zoomed-in box for comparison, middle: the zoomed-in boxes reconstructed with DF, right: the zoomed-in boxes reconstructed with BOSVS. The second row gives the results using zubal phantom data.

Since these experiments are based on Monte Carlo simulations, we can get the true activity distributions at any time exactly. In order to analyze the reconstruction results quantitatively, we define the measurements as follows:

$$\begin{array}{c} bias=\frac{1}{n}\sum_{i=1}^{n}\left(\frac{u_{i}-{\hat{u}}_{i}}{{\hat{u}}_{i}}\right), \end{array}$$ (20)

$$\begin{array}{c} variance=\frac{1}{n}\sum_{i=1}^{n}{\left(\frac{u_{i}-{\bar{u}}_{n}}{{\hat{u}}_{i}}\right)}^{2} \end{array}$$ (21)

where *u*_*i*_, ${\hat{u}}_{i}$ and ${\bar{u}}_{n}$ represent the estimated activity value of pixel i, the true activity value of pixel *i*, and the average estimated value of all the *n* pixels in one ROI respectively. Furthermore, we compute the contrast recovery coefficient (CRC), which is calculated by

$$\begin{array}{c} CRC=\frac{{\left(Contrast\right)}_{measure}}{{\left(Contrast\right)}_{theory}}=\frac{{\left(S/B\right)}_{measure}-1}{{\left(S/B\right)}_{theory}-1}, \end{array}$$ (22)

where *S* is the mean activity of the region of interest and *B* is the mean activity of the white matter region (background) in the reconstructed image. Fig 4 gives the true activity distributions and reconstructed images of the center slice by DF and BOSVS. Table 1 calculates the bias and variance between true activity distributions and reconstruction results by DF and BOSVS respectively. Fig 5 shows the regions of phantom and profile lines. And Fig 6 shows the profiles of reconstruction results by DF and BOSVS compared with the ground truth. Fig 7 gives the details of our selected area in the image reconstructed by two methods. We can obviously see that BOSVS show better recovery of activity distributions than DF.

**Table 1:** The calculated bias and variance of reconstruction results.

	brain phantom					
	All Region		ROI1		ROI2	
	bias	variance	bias	variance	bias	variance
DF	0.05594	0.06261	0.05889	0.04495	0.0987	0.05727
BOSVS	0.03513	0.04963	0.04855	0.03891	0.07239	0.05462
	zubal phantom					
	All Region		ROI1		ROI2	
	bias	variance	bias	variance	bias	variance
DF	0.08137	0.1013	0.1362	0.07379	0.1959	0.08583
BOSVS	0.03664	0.0663	0.1238	0.03893	0.0404	0.03960

#### Noise Toleration

**Fig 8:**
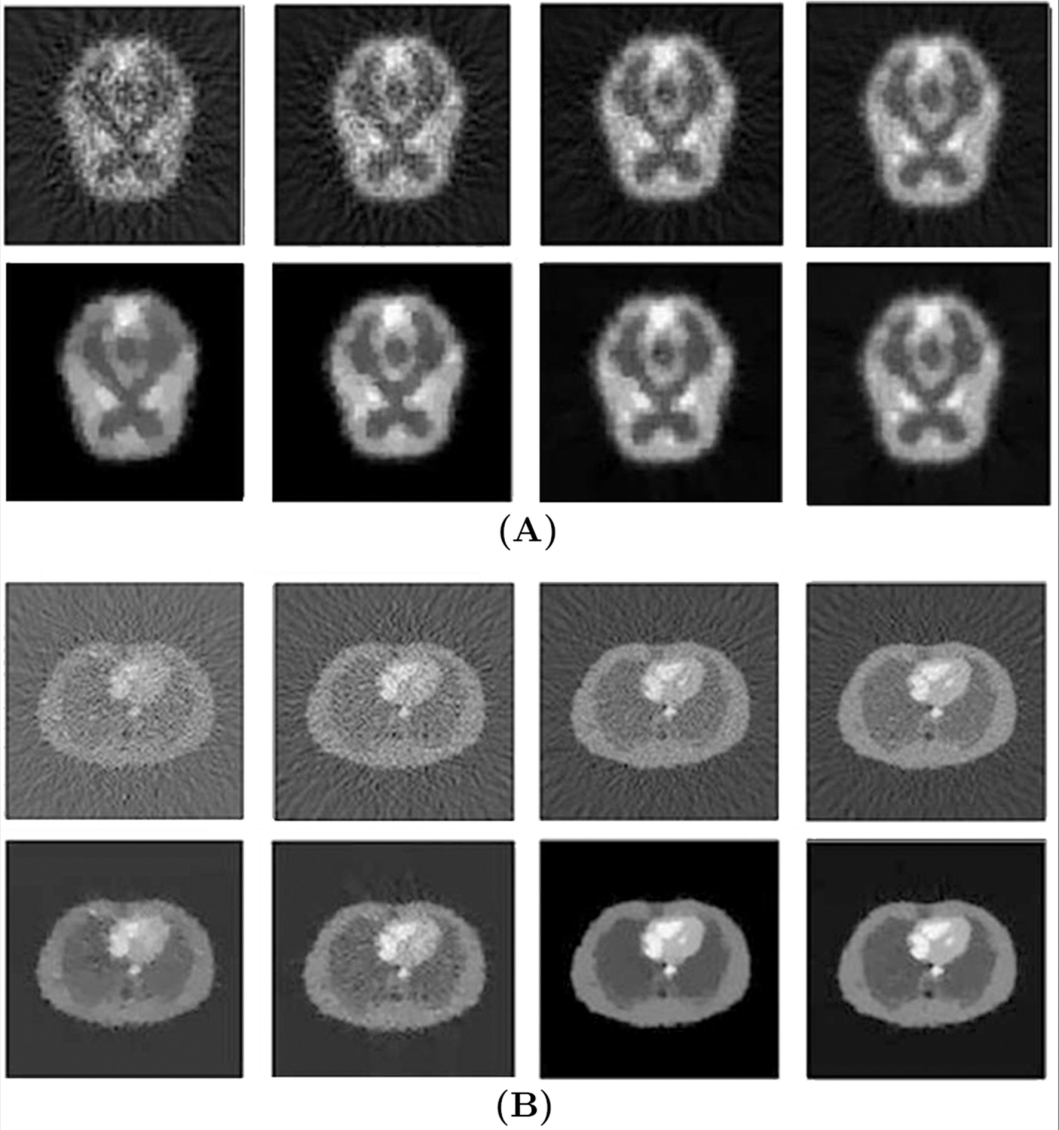
Reconstruction results. (A) Using brain phantom data, (B) Using zubal phantom data in different counting rates. The first row gives the results with DF, from left to right, the counting rate is 5 × 10^5^,1 × 10^6^,3 × 10^6^,9 × 10^6^. The second row gives the results with BOSVS.

We provide four Monte Carlo simulated sinogram for DF and BOSVS reconstruction in different kinds of counting rates and the results are illustrated as Fig 8. Obviously we can conclude that BOSVS results in a higher accuracy than DF. BOSVS can also have a good reconstructed result even in low counting rate.

**Fig 9:**
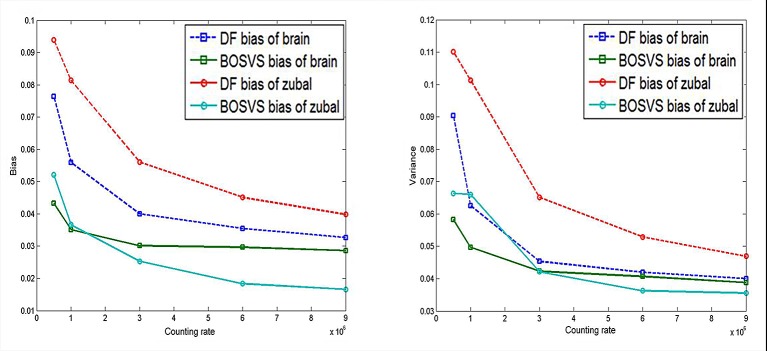
Calculated bias and variance. Left: bias of results reconstructed with DF and BOSVS versus counting rate. Right: variance of results reconstructed with DF and BOSVS versus counting rate.

The calculated bias and variance between reconstruction results and true activity distributions through all four kinds of counting levels are plotted in Fig 9, and some results are extracted and listed in Table 2 for better understanding. From the quantitative analysis in the experiment, the bias and variance fall with the counting rate increasing. Moreover, BOSVS has a better performance than DF whatever the counting rate is.

**Table 2:** The calculated bias and variance of reconstruction results in different counting rate.

	Counting rate	brain phantom				zubal phantom			
		bias		variance		bias		variance	
		DF	BOSVS	DF	BOSVS	DF	BOSVS	DF	BOSVS
All-Region	5.00E + 05	0.0765	**0.0433**	0.0904	**0.0583**	0.0940	0.0521	0.1101	0.0664
	1.00E + 06	0.0559	0.0351	0.0626	0.0496	0.0814	**0.0366**	0.1013	**0.0660**
	3.00E + 06	0.0400	0.0302	0.0453	0.0423	0.0561	0.0253	0.0651	0.0422
	6.00E + 06	0.0355	0.0297	0.0420	0.0407	0.0451	0.0184	0.0529	0.0363
	9.00E + 06	0.0327	0.0286	0.0401	0.0388	0.0398	0.0166	0.0469	0.0355
ROI1	5.00E + 05	0.0624	0.0569	0.0488	0.0406	0.2299	0.1988	0.0963	0.0444
	1.00E + 06	0.0589	**0.0486**	0.0450	**0.0389**	0.1362	0.1239	0.0738	0.0389
	3.00E + 06	0.0483	0.0438	0.0363	0.0351	0.0733	0.0488	0.0544	0.0350
	6.00E + 06	0.0431	0.0415	0.0312	0.0308	0.0538	**0.0273**	0.0417	**0.0332**
	9.00E + 06	0.0400	0.0388	0.0304	0.0292	0.0474	0.0225	0.0365	0.0309
ROI2	5.00E + 05	0.1070	**0.0884**	0.0689	**0.0564**	0.1994	0.0462	0.1159	0.0440
	1.00E + 06	0.0987	0.0723	0.0573	0.0546	0.1959	0.0404	0.0858	0.0435
	3.00E + 06	0.07224	0.0619	0.0483	0.0521	0.1146	0.0332	0.0712	0.0432
	6.00E + 06	0.05787	0.0539	0.0419	0.0451	0.0913	0.0270	0.0597	0.0432
	9.00E + 06	0.05674	0.05276	0.0412	0.0440	0.0792	**0.0225**	0.0552	**0.0396**
ROI3	5.00E + 05	0.1766	0.0757	0.1001	0.0524	0.2814	0.2095	0.1472	0.0951
	1.00E + 06	0.1150	0.0640	0.0798	0.0449	0.2802	0.1266	0.1163	0.0734
	3.00E + 06	0.0793	**0.0624**	0.0551	**0.0433**	0.1548	0.0596	0.0889	0.0600
	6.00E + 06	0.0711	0.0598	0.0464	0.0408	0.1171	0.0546	0.0711	0.0501
	9.00E + 06	0.0682	0.0576	0.0427	0.0388	0.1012	**0.0506**	0.0642	**0.0498**
ROI4	5.00E + 05	0.1922	0.0885	0.1067	0.0645	0.5123	0.1515	0.1619	0.1189
	1.00E + 06	0.1163	0.0622	0.0727	0.0454	0.5122	0.1370	0.1238	0.1055
	3.00E + 06	0.0752	**0.0613**	0.0520	**0.0445**	0.3108	0.1174	0.1087	0.0914
	6.00E + 06	0.0723	0.0573	0.0482	0.0435	0.2554	0.0751	0.0953	0.0813
	9.00E + 06	0.0650	0.0550	0.0465	0.0421	0.2194	**0.0721**	0.0891	**0.0808**
ROI5	5.00E + 05	0.3361	0.2219	0.1113	0.0848				
	1.00E + 06	0.2347	**0.1525**	0.0991	**0.0886**				
	3.00E + 06	0.1659	0.1492	0.0789	0.0759				
	6.00E + 06	0.1646	0.1378	0.0749	0.0758				
	9.00E + 06	0.1541	0.1289	0.0752	0.0742				

#### Comparison of Different ROIs

**Fig 10:**
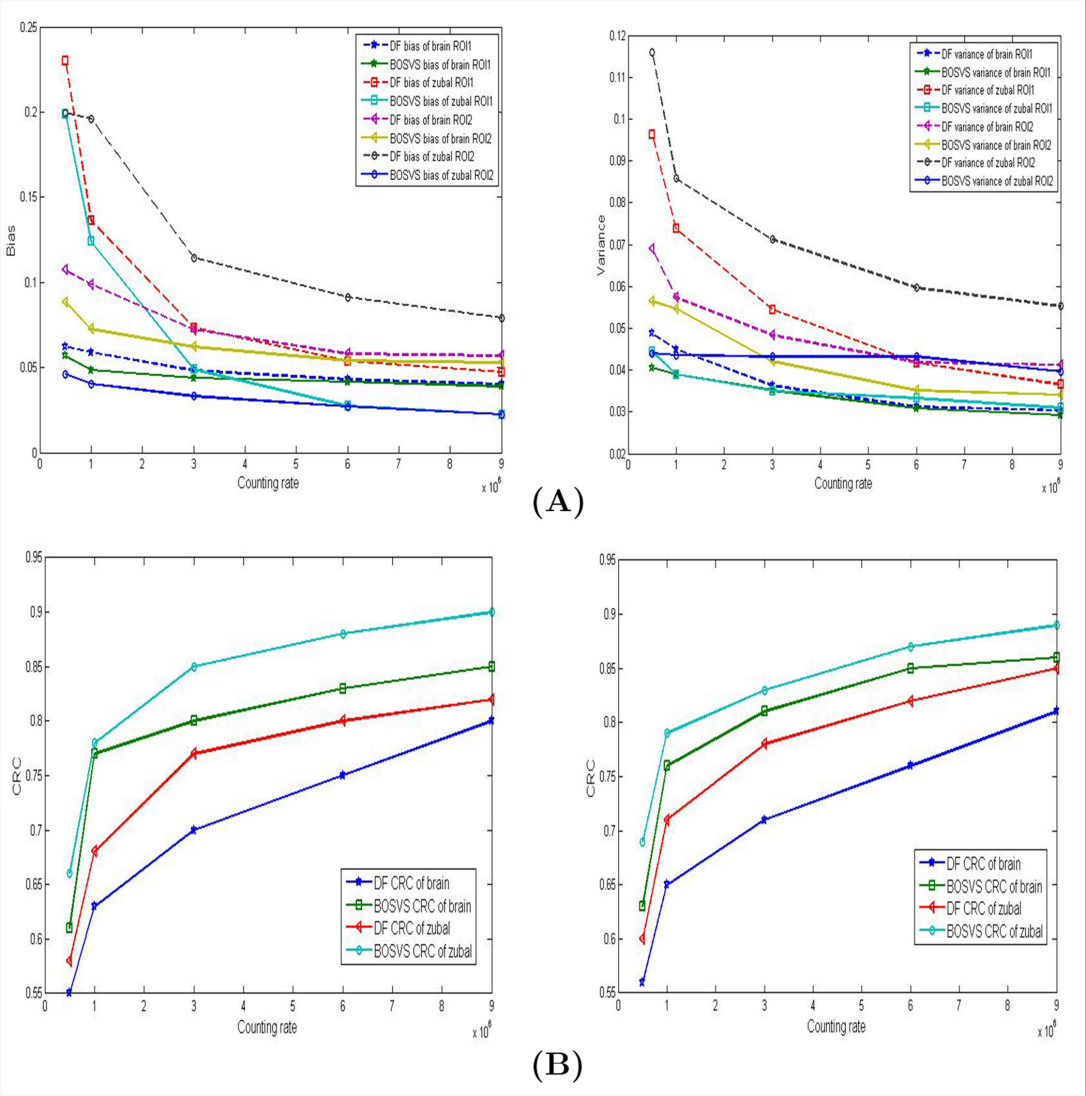
Results comparison in different ROIs. (A) Measurements (left: bias, right: variance) of ROIs marked in Fig 5 reconstructed with DF and BOSVS versus counting rate. (B) CRC of different ROIs (left: ROI1 right: ROI2) versus counting rate. Note that we take ROI1 and ROI2 for example.

Different regions in phantom may have different physiological and physical property, and reconstruction of a small target in the presence of background activity can be a challenge in PET imaging, as small targets may either be visually obstructed by noise in a regularized reconstruction or be over-smoothed by regularization. In this section we compare two reconstruction methods aimed at different regions. Take ROI1 and ROI2 for example, Fig 10(A) gives the image of bias and variance versus counting rate in different ROIs of phantom, and more details are shown in Table 2. Fig 10(B) compares the contrast recovery coefficient (CRC) versus counting rate by different methods for two regions of interest (ROI). The plots show that the proposed method achieves a better performance than DF in different regions, even sinogram is in low counting level.

#### Effect of Parameters

**Fig 11:**
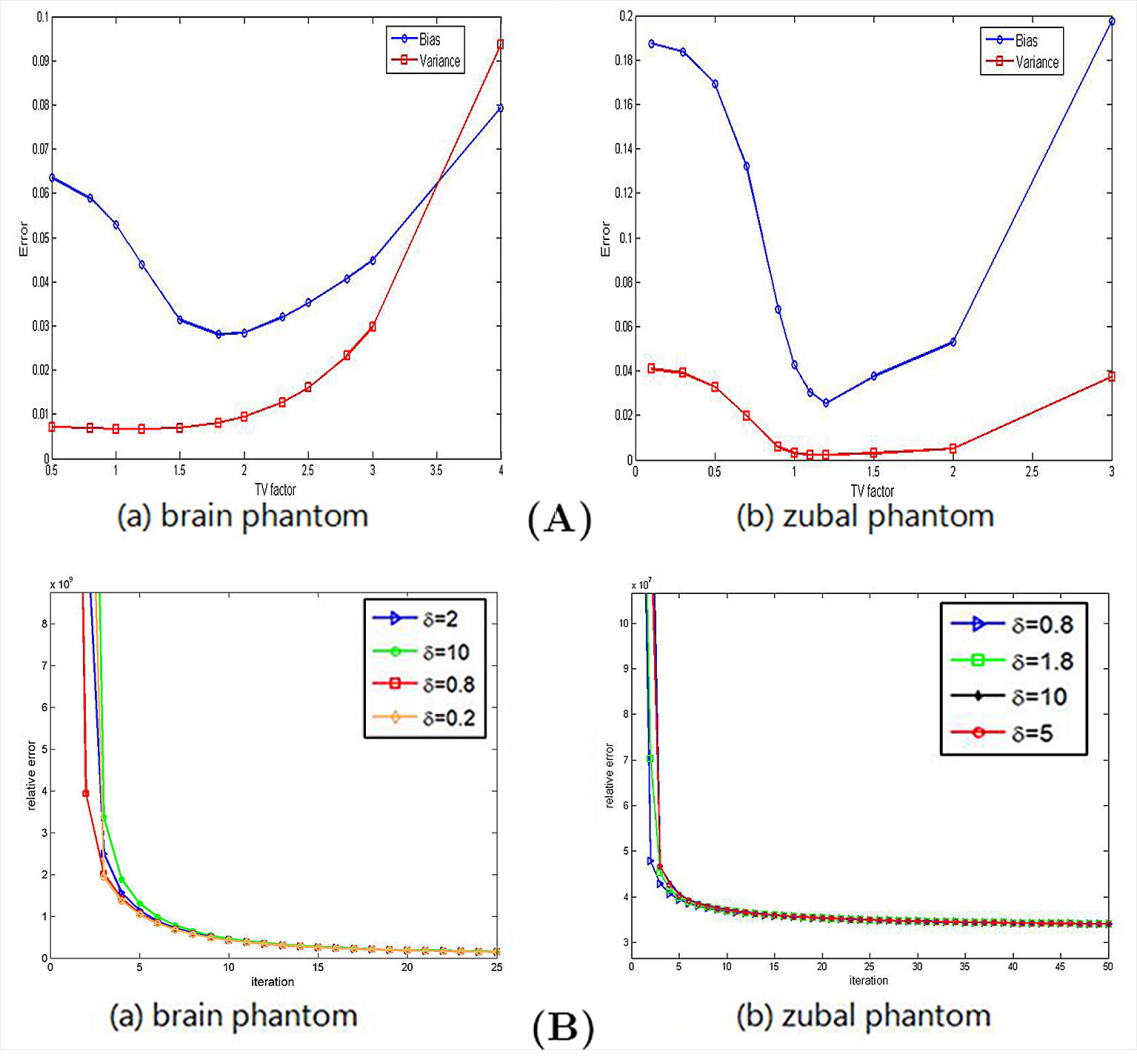
Parameter robustness. (A) Effect of TV factor. Left: bias and variance of results using brain phantom data versus TV weighting factor. Right: bias and variance of results using zubal phantom data versus TV weighting factor. (B) Effect of step size initialization. It illustrates the relative error versus iteration parameterized with different inial value of step size (left: brain phantom, right: zubal phantom).

From our proposed model, *α* is the weighting factor of TV, which can be optimized to smooth results. Fig 11(A) shows the bias and variance when TV factor *α* is changed. By increasing the TV weight factor, we can see that the bias and variance of reconstructed results decrease first and then after one optimal point, bias and variance will increase. As we know, TV can smooth the image, to get the balance of data fidelity and TV, we choose *α* = 1.5 for brain phantom and *α* = 1.1 for zubal phantom to get the best reconstructed results.

The other important parameter in the proposed model is the initialization of step size *δ*, Fig 11(B) gives the relative error between reconstruction results and true activity distributions versus iterations in BOSVS with different initialization of step size *δ*. We can see that the step size nearly has nothing to do with the convergence of the reconstruction algorithm. It just matters how fast the algorithm converge at the beginning, and at last the iterations will stop at the same time with all kinds of step size initialization. So in this experiment, we just choose the step size initialization *δ* as 0.8 both for brain phantom and Zubal phantom.

### Real Data

Real data experiments are performed on CTI ECAT data, and 47 slices real patient data are obtained. The scanner used is CTI ECAT PET system, which contains three rings of 48 block detectors each, for an axial field of view of 16.2 cm. The raw sinogram, along with the attenuation and efficiency factors, are radial samples by 192 angular samples (over 180 degrees), and will be reconstructed to images of 128*128. The values of sinogram range from 30.76 to 98.28, and sum is 1.75756 × 10^6^. Sinogram have been pre-corrected for delayed coincidences, and four slices of reconstructed images are presented for visual judgment. Note that all these data are publicly available in Fessler’s website. Patient records/information has been anonymized and de-identified prior to analysis.

**Fig 12:**
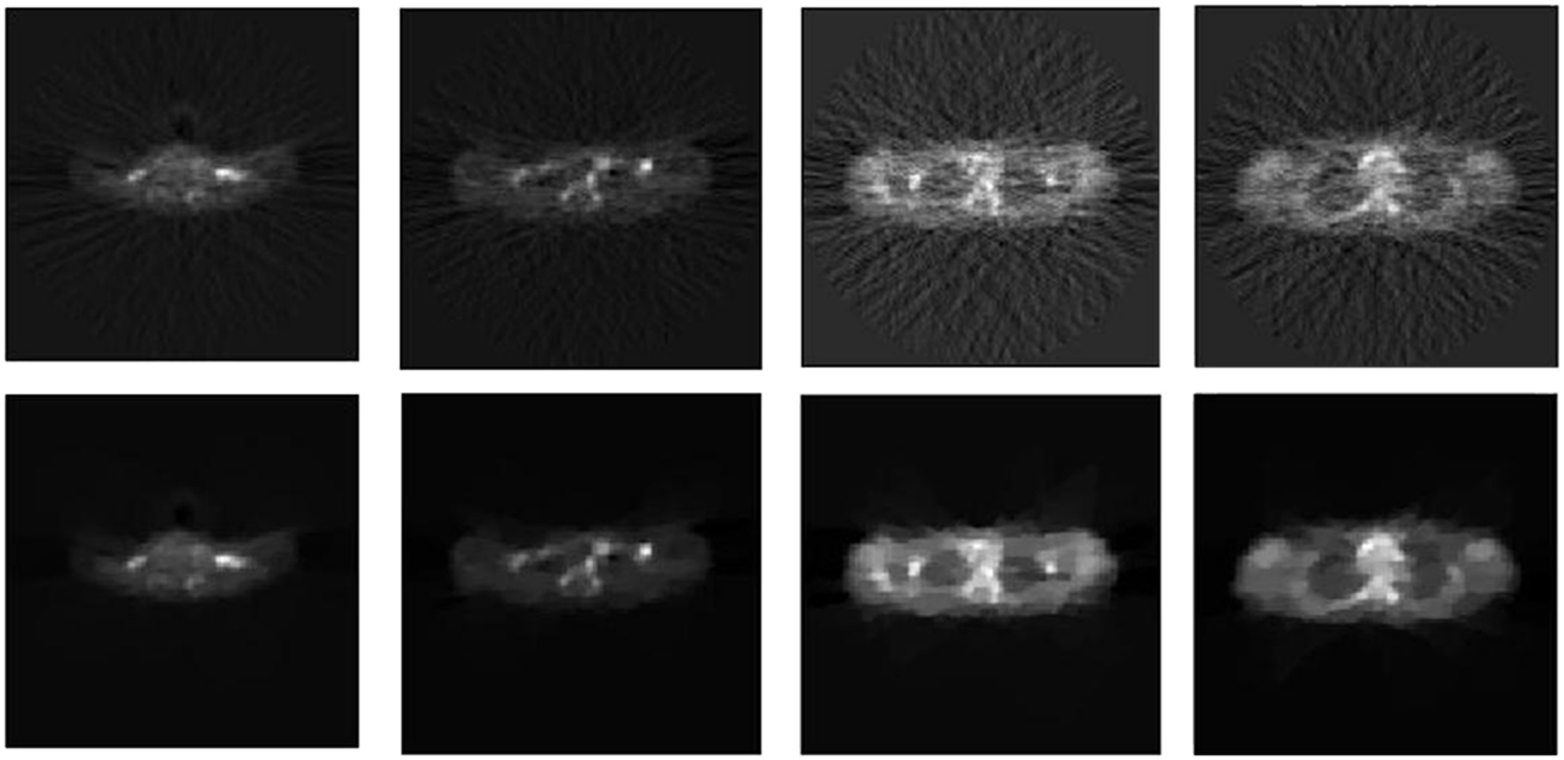
Image reconstruction with DF and BOSVS using four slices of CTI ECAT data. The upper row represents the results achieved from DF, while the bottom represents the results of BOSVS. From left to right, the slice of data is 19th, 27th, 35th and 43th slice.

Fig 12 shows the reconstruction results of all the four slices by DF (direct Fourier) and BOSVS. We can obviously see that BOSVS perform far better than traditional direct Fourier.

**Fig 13:**
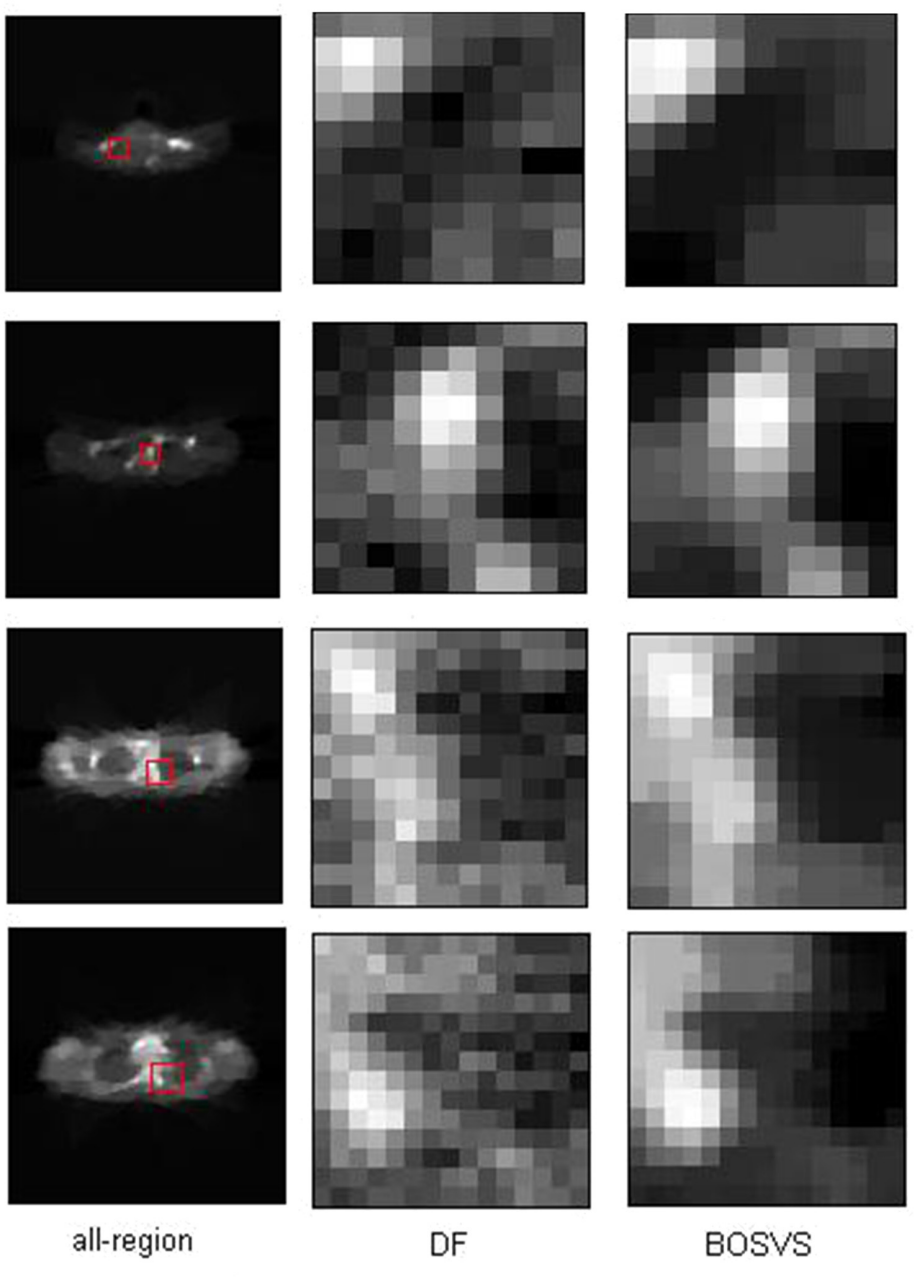
Selected area of reconstructed results. Each row shows results using one slice of data, left: reference image with red rectangle indicating the zoomed-in box for comparison, middle: the zoomed-in boxes reconstructed with DF, right: the zoomed-in boxes reconstructed with BOSVS. From up to down, the slice of data is 19th, 27th, 35th and 43th slice.

Fig 13 gives the details of our selected area in the image reconstructed by two methods, we mark out our interest area of all four slices with the red rectangles in the reconstruction result by BOSVS, then compare the details of the same area of reconstruction results by two methods, we can conclude that BOSVS shows better recovery of activity distributions than DF even in small regions.

## Discussion

This paper introduces total variation to traditional PET direct Fourier reconstruction methods to preserve the edges or boundaries more accurately and keep images more smooth. The main advantage of the proposed method is its capability of generating a high-resolution reconstruction results. We discuss the performance of the proposed method with DF in term of benefit of total variation regularization, performance of a small target or regions, initialization issues and robustness in the following context.

### Benefit of Total Variation Regularization

The experiments on Monte Carlo simulation data tested the reconstruction performance of traditional DF reconstruction method and the proposed method. Both in the brain phantom and zubal phantom, DF gives a relatively less bias and variance. In contrast, the proposed method exhibits high accuracy and stability, as indicated in Figs 4, 6, 7 and Table 1. Meanwhile, DF does not perform well when our data is in low counting rate, DF gives a poor bias and variance when the counting rate of data are far less than 1 × 10^6^, and DF even could not give an acceptable reconstruction results in low counting level. Whereas the bias and variance of our proposed method are far better as shown in the Table 2, total variation shows strong noise toleration and reconstruction performance.

The effectiveness of our method with total variation in estimating reconstruction performance is also extensively examined in the real data experiments. The reconstructed images indicate that the accuracy of results are greatly improved using our proposed method. Four slices real patient data, obtained from CTI ECAT PET system, with high level noises and kinds of physical factors, can be recovered with higher quality than DF reconstruction methods, as shown in Figs 12 and 13. It is clear that the results achieved through BOSVS have less background noise and present a much clearer contour than that of DF. However, it can be noticed that there are some rough color lumps which is very likely to appear when we add TV regularization into our reconstruction algorithm since TV based algorithms tend to over-smooth image details and textures. [51]

### Performance of a Small Target or Regions

We compare the reconstructed images by the DF and proposed methods in Fig 7 and we apply CRC as our measurement for reconstruction of regions. The image reconstructed by DF has a CRC of 0.55 for brain phantom and 0.58 for zubal phantom. In comparison, the BOSVS preserves the contrast (CRC: brain 0.61 zubal 0.66). And BOSVS demonstrates a more robust performance of CRC versus counting rate than DF. These results indicate that the proposed method can work robustly for reconstruction for both large and small targets.

### Initialization Issues

The number of iterations, the measurement, and the initialization of the external noise and all parameters are all important in our proposed methods. The measurements represent information about the examined object, but our method does not explicitly require the type of measurements. On the other hand, in order to acquire a strain image of high quality, we simply perform 100 iterations for the scheme.

The experiments on Monte Carlo simulation data and real patient data indicate that a convenient guideline for the initialization of parameter in proposed method can be easily concluded as follows:

- Since real patient data are originally noisy and we make kinds of experiments in different counting rates, the external noise can be initialized to zero.
- Most of the parameters of BOSVS such as *u*, *w*, *λ* always change with iterations, and BOSVS is almost an automated algorithm. The initialization of these parameters will not affect the reconstruction performance. So in our proposed methods, we initialize these parameters as 0, and the values of parameters will be adjusted by the algorithm with iterations.
- TV weighting factor *α* and the initialization of step size *δ* has been discussed in Fig 11. The initialization should be adjusted according to each case, and should be carefully set by the designer to obtain the desired estimation performance.

### Robustness of the Proposed Method

The proposed method can better deal with internal and external disturbances/noise, and only require a TV weighting factor and a initialization of step size for the iterative recovery process. It makes no assumptions about external noise statistics, which makes it more appropriate for certain practical problems where the disturbances/uncertainties are unknown. The robustness of the proposed method to noise was evaluated by employing different counting rate simulated sinogram data (For brain phantom, bias and variance of BOSVS are almost 60% of ones of DF), and we also examine the sensitivity of step size initialization for BOSVS(No matter what the initial value is, the iteration will stop at no more than 50th loop).

## Conclusion

This paper presents a total variation constrained direct Fourier reconstruction algorithm for 3D PET. By introducing TV regularization to traditional PET imaging model, we can enable smoother results and better denoising performance. Monte Carlo simulation and real patient data show the proposed method can implement the reconstruction in higher accuracy compared with traditional direct Fourier method, and it requires few iterations and little computational cost.

## References

[1] Valk PE. Positron emission tomography: basic sciences. Springer; 2003.

[2] Scheins J, Weirich C, Caldeira L, Lohmann P, Kops ER, Tellmann L, et al High-resolution, quantitative 3D PET image reconstruction for the Siemens hybrid 3T MR/BrainPET scanner using the PET reconstruction software toolkit (PRESTO). EJNMMI Physics. 2014;1(1):1–2. 26501640 10.1186/2197-7364-1-S1-A51 PMC4545227

[3] Zhou J, Qi J. Fast and efficient fully 3D PET image reconstruction using sparse system matrix factorization with GPU acceleration. Physics in medicine and biology. 2011;56(20):6739 10.1088/0031-9155/56/20/015 21970864 PMC4080908

[4] Schleyer PJ, O’Doherty MJ, Marsden PK. Extension of a data-driven gating technique to 3D, whole body PET studies. Physics in medicine and biology. 2011;56(13):3953 10.1088/0031-9155/56/13/013 21666288

[5] Defrise M, Kinahan PE, Townsend DW, Michel C, Sibomana M, Newport D. Exact and approximate rebinning algorithms for 3-D PET data. Medical Imaging, IEEE Transactions on. 1997;16(2):145–158. 10.1109/42.563660 9101324

[6] Bailey DL. 3D acquisition and reconstruction in positron emission tomography. Annals of nuclear medicine. 1992;6(3):123–130. 10.1007/BF03178303 1389886

[7] Sugawara S, Nakamura K, Kinoshita F, Kinoshita T, Ibaraki M. The effect of activity outside the field-of-view on image signal-to-noise ratio for 3D PET with 15 O. Physics in Medicine and Biology. 2011;56(10):3061–3072. 10.1088/0031-9155/56/10/011 21508441

[8] Bai B, Smith AM. Fast 3D iterative reconstruction of PET images using PC graphics hardware. In: Nuclear Science Symposium Conference Record, 2006. IEEE. vol. 5. IEEE; 2006. p. 2787–2790.

[9] Kim KS, Ye JC. Fully 3D iterative scatter-corrected OSEM for HRRT PET using a GPU. Physics in medicine and biology. 2011;56(15):4991 10.1088/0031-9155/56/15/021 21772080

[10] Magdics M, Szirmay-Kalos L, Tóth B, Légrády D, Cserkaszky A, Balkay L, et al. Performance evaluation of scatter modeling of the GPU-based “Tera-Tomo” 3D PET reconstruction. In: Nuclear Science Symposium and Medical Imaging Conference (NSS/MIC), 2011 IEEE. IEEE; 2011. p. 4086–4088.

[11] Bai B, Lin Y, Zhu W, Ren R, Li Q, Dahlbom M, et al MAP reconstruction for Fourier rebinned TOF-PET data. Physics in medicine and biology. 2014;59(4):925 10.1088/0031-9155/59/4/925 24504374 PMC3980855

[12] Defrise M, Geissbuhler A, Townsend D. A performance study of 3D reconstruction algorithms for positron emission tomography. Physics in medicine and biology. 1994;39(3):305 10.1088/0031-9155/39/3/001 15551582

[13] Chatziioannou A, Qi J, Moore A, Annala A, Nguyen K, Leahy R, et al Comparison of 3-D maximum a posteriori and filtered backprojection algorithms for high-resolution animal imaging with microPET. Medical Imaging, IEEE Transactions on. 2000;19(5):507–512. 10.1109/42.870260 11021693

[14] Frese T, Rouze NC, Bouman CA, Sauer K, Hutchins GD. Quantitative comparison of FBP, EM, and Bayesian reconstruction algorithms for the IndyPET scanner. Medical Imaging, IEEE Transactions on. 2003;22(2):258–276. 10.1109/TMI.2002.808353 12716002

[15] De Francesco S, da Silva AMF. Efficient NUFFT-based direct Fourier algorithm for fan beam CT reconstruction. In: Medical Imaging 2004. International Society for Optics and Photonics; 2004. p. 666–677.

[16] Defrise M, Panin VY. System and method for 3D time of flight PET forward projection based on an exact axial inverse rebinning relation in fourier space. Google Patents; 2011. US Patent 8,000,513.

[17] Ahn S, Cho S, Li Q, Lin Y, Leahy RM. Optimal rebinning of time-of-flight PET data. Medical Imaging, IEEE Transactions on. 2011;30(10):1808–1818. 10.1109/TMI.2011.2149537 PMC3353661 21536530

[18] Delso G, Fürst S, Jakoby B, Ladebeck R, Ganter C, Nekolla SG, et al Performance measurements of the Siemens mMR integrated whole-body PET/MR scanner. Journal of nuclear medicine. 2011;52(12):1914–1922. 10.2967/jnumed.111.092726 22080447

[19] Kinahan PE, Rogers J. Analytic 3D image reconstruction using all detected events. Nuclear Science, IEEE Transactions on. 1989;36(1):964–968. 10.1109/23.34585

[20] Matej S, Lewitt RM. 3D-FRP: direct Fourier reconstruction with Fourier reprojection for fully 3-D PET. Nuclear Science, IEEE Transactions on. 2001;48(4):1378–1385. 10.1109/23.958359

[21] Knešaurek K. Fourier-wavelet restoration in PET/CT brain studies. Nuclear Instruments and Methods in Physics Research Section A: Accelerators, Spectrometers, Detectors and Associated Equipment. 2012;689:29–34. 10.1016/j.nima.2012.06.032

[22] Watson C. New, faster, image-based scatter correction for 3D PET. Nuclear Science, IEEE Transactions on. 2000;47(4):1587–1594. 10.1109/23.873020

[23] Surti S, Kuhn A, Werner ME, Perkins AE, Kolthammer J, Karp JS. Performance of Philips Gemini TF PET/CT scanner with special consideration for its time-of-flight imaging capabilities. Journal of Nuclear Medicine. 2007;48(3):471–480. 17332626

[24] Zaidi H, Ojha N, Morich M, Griesmer J, Hu Z, Maniawski P, et al Design and performance evaluation of a whole-body Ingenuity TF PET–MRI system. Physics in medicine and biology. 2011;56(10):3091 10.1088/0031-9155/56/10/013 21508443 PMC4059360

[25] Daube-Witherspoon ME, Matej S, Werner ME, Surti S, Karp JS. Comparison of list-mode and DIRECT approaches for time-of-flight PET reconstruction. Medical Imaging, IEEE Transactions on. 2012;31(7):1461–1471. 10.1109/TMI.2012.2190088 PMC3389166 22410326

[26] Tong S, Alessio AM, Kinahan PE. Image reconstruction for PET/CT scanners: past achievements and future challenges. Imaging in medicine. 2010;2(5):529–545. 10.2217/iim.10.49 21339831 PMC3039307

[27] Razifar P, Sandström M, Schnieder H, Långström B, Maripuu E, Bengtsson E, et al Noise correlation in PET, CT, SPECT and PET/CT data evaluated using autocorrelation function: a phantom study on data, reconstructed using FBP and OSEM. BMC medical imaging. 2005;5(1):5 10.1186/1471-2342-5-5 16122383 PMC1208889

[28] Dutta J, Ahn S, Li C, Cherry SR, Leahy RM. Joint L1 and total variation regularization for fluorescence molecular tomography. Physics in medicine and biology. 2012;57(6):1459 10.1088/0031-9155/57/6/1459 22390906 PMC3380088

[29] Ueda S, Nakamiya N, Matsuura K, Shigekawa T, Sano H, Hirokawa E, et al Optical imaging of tumor vascularity associated with proliferation and glucose metabolism in early breast cancer: clinical application of total hemoglobin measurements in the breast. BMC cancer. 2013;13(1):514 10.1186/1471-2407-13-514 24176197 PMC3817816

[30] Pauleit D, Floeth F, Tellmann L, Hamacher K, Hautzel H, Müller HW, et al Comparison of O-(2–18F-fluoroethyl)-L-tyrosine PET and 3–123I-iodo-*α*-methyl-L-tyrosine SPECT in brain tumors. Journal of Nuclear Medicine. 2004;45(3):374–381. 15001676

[31] Seemann M, Meisetschlaeger G, Gaa J, Rummeny E. Assessment of the extent of metastases of gastrointestinal carcinoid tumors using whole-body PET, CT, MRI, PET/CT and PET/MRI. European journal of medical research. 2006;11(2):58 16504962

[32] Somayajula S, Panagiotou C, Rangarajan A, Li Q, Arridge SR, Leahy RM. PET image reconstruction using information theoretic anatomical priors. Medical Imaging, IEEE Transactions on. 2011;30(3):537–549. 10.1109/TMI.2010.2076827 PMC3331595 20851790

[33] Asma E, Ahn S, Qian H, Gopalakrishnan G, Thielemans K, Ross SG, et al. Quantitatively Accurate Image Reconstruction for Clinical Whole-Body PET Imaging. 2012;.

[34] Hawe D, Hernández Fernández FR, O’Suilleabháin L, Huang J, Wolsztynski E, O’Sullivan F. Kinetic analysis of dynamic positron emission tomography data using open-source image processing and statistical inference tools. Wiley Interdisciplinary Reviews: Computational Statistics. 2012;4(3):316–322. 10.1002/wics.1196 23087780 PMC3472445

[35] Mishra A, Wong A, Clausi DA, Fieguth P. A Bayesian Information Flow Approach to Image Segmentation. In: Computer and Robot Vision (CRV), 2010 Canadian Conference on. IEEE; 2010. p. 301–308.

[36] Rudin LI, Osher S, Fatemi E. Nonlinear total variation based noise removal algorithms. Physica D: Nonlinear Phenomena. 1992;60(1):259–268. 10.1016/0167-2789(92)90242-F

[37] Fessler JA, Booth SD. Conjugate-gradient preconditioning methods for shift-variant PET image reconstruction. Image Processing, IEEE Transactions on. 1999;8(5):688–699. 10.1109/83.760336 18267484

[38] Kaufman L, Neumaier A. PET regularization by envelope guided conjugate gradients. Medical Imaging, IEEE Transactions on. 1996;15(3):385–389. 10.1109/42.500147 18215919

[39] Rapisarda E, Presotto L, De Bernardi E, Gilardi MC, Bettinardi V. Optimized Bayes variational regularization prior for 3D PET images. Computerized Medical Imaging and Graphics. 2014;. 10.1016/j.compmedimag.2014.05.004 24958594

[40] Jonsson E, Huang Sc, Chan T. Total variation regularization in positron emission tomography. CAM report. 1998;p. 98–48.

[41] Kisilev P, Zibulevsky M, Zeevi YY. Wavelet representation and total variation regularization in emission tomography. In: Image Processing, 2001. Proceedings. 2001 International Conference on. vol. 1. IEEE; 2001. p. 702–705.

[42] Burger M, Müller J, Papoutsellis E, Schönlieb CB. Total Variation Regularisation in Measurement and Image space for PET reconstruction. arXiv preprint arXiv:14031272. 2014;.

[43] Lu L, Hu D, Han Y, Gu C, Rahmim A, Ma J, et al Partial volume correction in small animal PET imaging incorporating total variation regularization In: Society of Nuclear Medicine Annual Meeting Abstracts. vol. 55 Soc Nuclear Med; 2014 p. 374.

[44] Kinouchi S, Yamaya, T Tashima H, Yoshida E, Ito H, Suga M. Total variation minimization for in-beam PET image reconstruction. In: Nuclear Science Symposium and Medical Imaging Conference (NSS/MIC), 2012 IEEE. IEEE; 2012. p. 3412–3414.

[45] Ye X, Chen Y, Huang F. Computational acceleration for MR image reconstruction in partially parallel imaging. Medical Imaging, IEEE Transactions on. 2011;30(5):1055–1063. 10.1109/TMI.2010.2073717 20833599

[46] Zhang X, Burger M, Osher S. A unified primal-dual algorithm framework based on Bregman iteration. Journal of Scientific Computing. 2011;46(1):20–46. 10.1007/s10915-010-9408-8

[47] Chen Y, Hager WW, Yashtini M, Ye X, Zhang H. Bregman operator splitting with variable stepsize for total variation image reconstruction. Computational Optimization and Applications. 2013;54(2):317–342. 10.1007/s10589-012-9519-2

[48] Defrise M, Liu X. A fast rebinning algorithm for 3D positron emission tomography using John’s equation. Inverse Problems. 1999;15(4):1047 10.1088/0266-5611/15/4/314

[49] Xu J, Dehaghani AR, Gao F, Wang L. A Novel Total Variation Based Noninvasive Transmural Electrophysiological Imaging In: Medical Image Computing and Computer-Assisted Intervention–MICCAI 2013. Springer; 2013 p. 501–508. 10.1007/978-3-642-40811-3_63 24505704

[50] Zubal IG, Harrell CR, Smith EO, Rattner Z, Gindi G, Hoffer PB. Computerized three-dimensional segmented human anatomy. Medical physics. 1994;21(2):299–302. 10.1118/1.597290 8177164

[51] Zhang J, Liu S, Xiong R, Ma S, Zhao D. Improved total variation based image compressive sensing recovery by nonlocal regularization. In: Circuits and Systems (ISCAS), 2013 IEEE International Symposium on. IEEE; 2013. p. 2836–2839.

